# A Digital Sexual Health Intervention for Urban Adolescent and Young Adult Male Emergency Department Patients: User-Centered Design Approach

**DOI:** 10.2196/55815

**Published:** 2024-10-04

**Authors:** Lauren S. Chernick, Mona Bugaighis, Victoria Daylor, Daniel Hochster, Evan Rosen, Rebecca Schnall, Melissa S Stockwell, David L. Bell

**Affiliations:** 1 Columbia University Irving Medical Center New York, NY United States; 2 Dom and Tom New York, NY United States

**Keywords:** sexual health, adolescent health, sex education, emergency medicine, health planning, sexual behavior, SMS text messaging, mHealth, mobile app, condom use, user-centered design

## Abstract

**Background:**

Adolescents and young adults frequently present to the emergency department (ED) for medical care and continue to have many unmet sexual health needs. Digital interventions show promise to improve adolescent and young adult sexual health; yet, few interventions focus on male ED patients, despite their infrequent use of contraceptives and rising rates of sexually transmitted infections.

**Objective:**

This paper describes the design and development of Dr. Eric (Emergency Room Interventions to Improve Care), a digital app focused on promoting condom use among sexually active adolescent and young adult male ED patients.

**Methods:**

This study followed 4 phases of app development, which were based on user-centered design and the software development lifecycle. In phase 1, define, we explored our target population and target health problem (infrequent condom use among male ED patients) by collecting key stakeholder input and conducting in-depth interviews with male patients and urban ED medical providers. In phase 2, discover, we partnered with a digital product agency to explore user experience and digital strategy. In phase 3, design, we refined Dr. Eric’s content, a 5-part sexual health educational module and a 10-week SMS text messaging program that focuses on condom use and partner communication about effective contraceptives. We conducted semistructured interviews with male adolescent and young adults to gather feedback on the app and perform usability testing, editing the app after each interview. We also interviewed informatics experts to assess the usability of a high-fidelity prototype. Interviews were recorded and analyzed via descriptive thematic analysis; informatic expert feedback was categorized by Nielsen’s heuristic principles. In phase 4, develop, we created the technical architecture and built a responsive web app. These findings were gathered leading to the final version of the digital Dr. Eric program.

**Results:**

Using data and key stakeholder input from phases 1 and 2, we iteratively created the Dr. Eric prototype for implementation in the ED setting. Interviews with 8 adolescent and young adult male ED patients suggested that users preferred (1) straightforward information, (2) a clear vision of the purpose of Dr. Eric, (3) open-ended opportunities to explore family planning goals, (4) detailed birth control method information, and (5) games presenting novel information with rewards. Five usability experts provided heuristic feedback aiming to improve the ease of use of the app. These findings led to the final version of Dr. Eric.

**Conclusions:**

Following these mobile health development phases, we created a digital sexual health mobile health intervention incorporating the principles of user experience and interface design. Dr. Eric needs further evaluation to assess its efficacy in increasing condom use among adolescent and young adult male ED patients. Researchers can use this framework to form future digital health ED-based digital interventions.

## Introduction

With over 20 million adolescents and young adults visiting emergency departments (EDs) each year in the United States, the ED visit represents a unique opportunity for preventive health intervention; yet, how to design interventions that can be implemented successfully into the distinct acute care setting remains unclear [1-3]. In the ED, wait times can be long, resources are limited, and medical providers are busy [4]. Providers often attend to the sickest of patients, sometimes at the expense of preventive conversations, to maintain patient flow [5,6]. ED-based interventions must recognize these barriers to not only be feasible and acceptable to both providers and patients but also sustain a chance of being implemented effectively within the ED system [7].

Although adolescents and young adults present for a variety of chief complaints, adolescent and young adult male ED patients continue to experience unmet needs leading to disparities for this younger population in sexual health outcomes [8,9]. Adolescent patients in urban EDs admit to infrequent condom use, with rates as low as 40% at last intercourse [10]. Adolescent detection rates in urban ED for sexually transmitted infection (STI) are as high as 26% depending on symptomatology [11-14]. Condom nonuse among this patient population has also been associated with other risky behaviors, including alcohol use, violence, and drug abuse [15,16]. Yet, in national studies, for many of these adolescents and young adults, the ED is their only source of medical care, and their communication with outpatient medical providers around sexual health topics is poor, especially regarding contraceptives [17,18]. The ED visit remains an important opportunity for intervention; yet, few exist in this setting [3].

Recent evidence suggests that digital interventions can lead to positive changes in adolescent and young adult sexual health; yet, few studies are designed with the complicated acute care setting in mind [19]. Digital interventions can incorporate tailored advice, goal setting, and feedback like face-to-face conversations [20]. In a recent meta-analysis, youth-focused, technology-based interventions were noted to improve key sexual behaviors, such as condom use; yet, of 16 studies included, only 1 specifically targeted male patients, despite adolescent and young adult male patients exhibiting poor knowledge of effective contraceptives, reporting few conversations about effective contraceptives with medical providers and sexual partners, and perceiving effective contraception as being outside of their locus of control [21]. In the ED setting specifically, digital interventions have focused on female contraception use [22-24]. These digital interventions aim to fit easily into the busy ED workflow by minimizing the time commitment of the ED provider while providing real-time evidence-based education to patients at high risk. Yet, to date, no ED-based digital intervention exists in the ED setting that focuses on adolescent and young adult male sexual health.

Considering the distinctiveness of the ED setting and the unmet sexual health needs of our adolescent and young adult ED population, our multidisciplinary team developed Dr. Eric (Emergency Room Interventions to Improve Care), a digital, evidence-based intervention focused on improving the sexual health of adolescent and young adult male patients seeking care in an urban New York City ED. We hypothesized that a mobile health (mHealth) app targeting adolescent and young adult male ED patients could deliver necessary preventive sexual health services that these patients are not receiving elsewhere. In this paper, we describe the phases followed to create the multimedia platform that integrated user-centered design principles, the collaboration between an academic team and a digital agency, and an iterative development methodology specific to the ED setting.

## Methods

### Study Design

**Figure 1:**
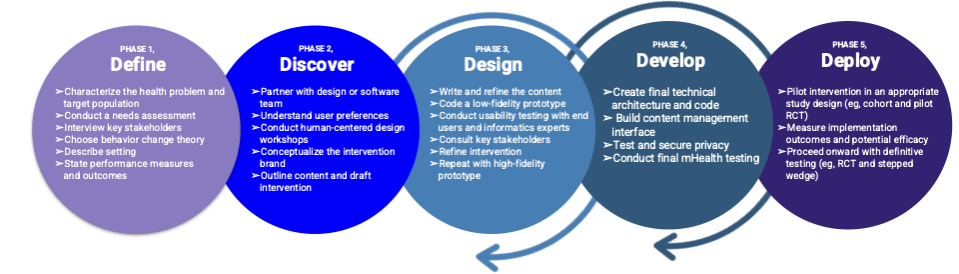
A flow diagram of the 5 phases followed to create theory-based, user-informed mHealth interventions. This paper describes the use of phases 1, 2, 3, and 4. mHealth: mobile health; RCT: randomized controlled trial.

This paper describes the 4 phases followed for app production: define, discover, design, and develop. A fifth phase, deploy, is ongoing. This framework was based on user-centered design, design thinking, the Lean approach, agile techniques (sprints), and usability testing [25-27]. Below each phase is described in greater detail and summarized in Figure 1.

### Phase 1: Define

As part of a needs assessment, we collected data from (1) key stakeholders (eg, informal interviews with 3 New York City high school teachers, 2 New York City health educators, and 1 social worker via collaboration with the New York City school-based health centers and the family planning clinic); (2) national and New York State sexual health curricula; (3) evidence-based sexual education guidelines; and (4) an extensive literature review, including experience implementing sexual health digital interventions in our ED [22,24,28,29]. We chose the social cognitive theory and motivational interviewing to inform our intervention to support adolescent contraceptive behavior change [30-33]. These findings were compiled into a conceptual model.

To better understand the context with whom we aimed to focus the intervention and its implementation in the acute care ED setting, we conducted pilot work in the form of qualitative interviews on 2 populations [5,34,35]. In one series of interviews, we spoke with adolescent and young adult male patients seeking care in our New York City ED to explore our targeted health problem (infrequent condom use) [34,35]. We also conducted 38 semistructured interviews with health care providers (ED attendings, nurses, and advanced care providers) across 5 national urban academic centers to better assess barriers and facilitators to providing confidential care and implementing preventive interventions in the ED setting. These interviews were complemented with site observations to understand ED workflows better [5].

### Phase 2: Discover

In the second phase, we partnered with a digital product agency and experts in user experience design and digital strategy [36]. Together, we worked to generate a shared understanding of what a digital male-focused sexual health intervention might function and look like in the ED setting. To better understand the digital preferences of our target population, we created a database of popular websites and apps focused on sexual health. Next, we proceeded through a series of design thinking workshops, where we developed patient personas and constructed a journey map of the intervention, showing a timeline of interactions the users would have with the program, starting with the ED and continuing into everyday life [27,37]. During this phase, we also created a list of user stories based on the prototype from phase 1, describing what would happen on each page of the intervention. These ED user stories defined the app experience for subsequent phases. After creating the brand of the intervention, the app content was laid out and designed as a low-fidelity prototype in Figma, a platform for creating, sharing, and testing of digital designs [38].

### Phase 3: Design

#### Overview

In our third phase, we designed and iterated the content of a 2-part intervention—an app consisting of 5 educational modules, followed by 10 weeks of once-a-week 2-way SMS text messages. The design team created templates based on the different types of content to create a high-fidelity prototype of the app experience. We also mapped out our outcomes and evaluation plan ensuring that needed data were captured by the app or external sources. All usability testing was approved by our local institutional review board.

To conclude our design phase, we tested the prototype to identify usability issues. For any new system to have true value and impact among its intended audience, a critical first step is to establish its usability [39,40]. The goals of usability testing are to identify potential problems with using the system, improve system design, and increase the likelihood of technology acceptance among end users [39]. To do this, we conducted user and informatics expert interviews. After each round of testing, we analyzed feedback and made updates to the design and content of the prototype.

#### Adolescent and Young Adult Male ED Patients

The goal of usability testing with end users is to understand how real users interact with a product to improve its design.

#### Participants

We recruited male adolescent and young adults who are ED patients aged 14-21 years who had been sexually active with female patients in the past 3 months. The majority of the population seeking care at our academic medical center identifies as predominantly Hispanic, is publicly insured, and lives in the local New York City regions of Washington Heights and the Bronx. Exclusion criteria included not owning a mobile phone, being too ill, having cognitive impairment, not speaking English, or wanting their partner to become pregnant in the next year [41]. Enrollment spanned an adult and pediatric ED.

#### Procedures and Data Analysis

Medical providers assessed potential eligibility. Research team members approached patients to verify eligibility and obtain informed consent. We obtained a waiver of parental consent for those participants younger than 18 years of age. In the ED, participants were provided with use cases for using Dr. Eric, interacted with the prototype on an ED tablet, and were asked to perform tasks that should closely mirror the intended end use of the app. Similar to procedures used in prior work, participants used a think-aloud method, in which they described what they were thinking, seeing, and trying to do as they performed the tasks required in the use cases [39]. The sessions were audio-recorded. After evaluation of the Dr. Eric app, participants also read aloud Dr. Eric SMS text messages sent to the tablet and provided qualitative feedback regarding their clarity, content, and likability. To analyze the interviews, 2 authors (LC and MB) used recordings and notes from the session to perform content analysis [42]. Comments and recommendations were categorized according to sections of the app. This iterative process involved testing the system and then using the findings to change it to better meet users’ needs.

#### Heuristic Experts

Heuristic evaluation is a usability inspection method for computer software or apps that help identify problems in user interface design [43,44].

#### Participants

We recruited 5 informaticians as usability experts via personal correspondence within our academic institution. All provided informed consent. Experts have training in human-computer interaction and published in the field of informatics.

#### Procedures and Data Analysis

Over videoconference, the usability experts shared a screen of the high-fidelity version of the Dr. Eric app. In addition, using the think-aloud method, evaluators’ comments about usability problems were categorized based on the heuristic principles [44].

### Phase 4: Develop

During this phase, the digital agency created the technical architecture and coded the web app based on the annotated designs and feedback. Because of the limited user base and scope, the intervention was coded as a responsive web app rather than a native app. We also built a content management interface where the team can easily edit intervention content and see and export responses from users who went through the intervention. During this phase, we also implemented an SMS texting campaign.

### Ethical Considerations

This qualitative component of this study was evaluated and approved by the Columbia University Institutional Review Board (AAAN4509). We collected no personally identifiable information; interviews remained anonymous. All participants provided informed consent via an information sheet; we received a waiver of informed consent from parents for participants younger than 18 years of age. Adolescent participants received a US $20 gift card, and usability experts received a US $100 gift card as compensation.

## Results

### Overview

After the define phase, we conducted 2 portions of discovery meetings, one before and one after the attainment of funding. During the design phase, interviews with adolescent and young adult patients and informatics experts were conducted in the winter and spring of 2021. App design, branding, language, and usability were edited iteratively based on the interviews.

### Phase 1: Define

**Figure 2:**
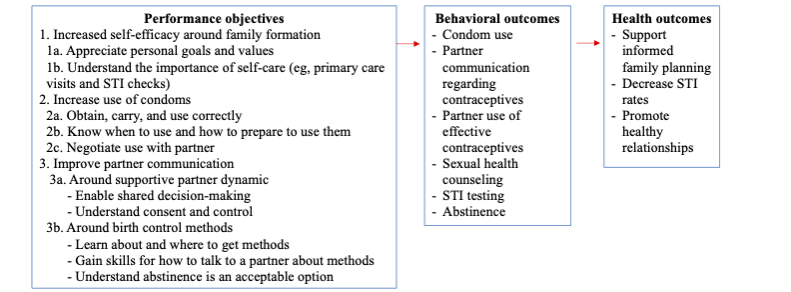
Our conceptual model of behavioral change with our ultimate health outcomes to improve adolescent and young adult male sexual health. STI: sexually transmitted infection.

Data from the first phase led to the creation of a logic model of change as seen in Figure 2.

Pilot interviews with adolescent and young adult male patients and ED providers elicited many key insights, which were previously published [5,34,35]. In summary, adolescent and young adult participants elicited the barriers and enablers affecting their condom use that ranged from individual factors (eg, perceived gender roles, trust in and control of condoms, and pregnancy intentions) to interpersonal factors (eg, partner communication about contraceptives and partner relationship and length) to community and societal factors (eg, school-based learning and access to contraception). We also explored their receptivity and preferences for sexual health interventions in the ED. While adolescents and young adults remained split on whether the intervention should target those presenting for a sexual health–related chief complaint versus be available to all ED patients, we found that these male patients viewed the ED visit as an unused time suitable for digital sexual health interventions that provided novel reliable and relatable information, allowed for user control, and maintained confidentiality. After the ED visit, they expressed how they would be less likely to engage with a program. In interviews with ED health care providers, key themes included how space, such as stretchers in the hallways, can act as a barrier to talking to adolescents alone and how having multiple patients who are sick at once can hinder their ability to provide comprehensive medical care. However, overall providers favored digital preventive health interventions as a way to minimize their burden of work yet still provide holistic care, which supported their professional identity. They recommended ways to ease implementation. Such ideas included how to increase motivation among those implementing the interventions, such as training study champions, as well as how to weave the intervention during ED “down time” such as implementing while patients wait for radiology tests or blood results.

### Phase 2: Discover

We built a database and categorized popular sexual health websites and apps based on tone (funny vs serious) as well as the media modality used to educate on contraceptive methods. Examples included engaging videos from the Centers for Disease Control and Prevention (CDC) and data visualizations on birth control methods from Bedsider [45,46]. Next, we constructed personas, which are profiles of potential ED users, based on the experiences and attitudes of participants from the semistructured qualitative interventions described in phase 1. Examples of ED patients included a 19-year-old male who was resistant to displaying vulnerability to gaps of sexual health knowledge and a 15-year-old male who worried about privacy as his parents peered over their shoulder during the ED visit. Personas helped focus the design process on our target population and how their unique outlook and personal history impact their perception of the app experience. Next, we created our journey map, which allowed us to explore the “feelings and mindsets” of the patients to appreciate a range of emotions and better match the intervention to experience.

**Figure 3:**
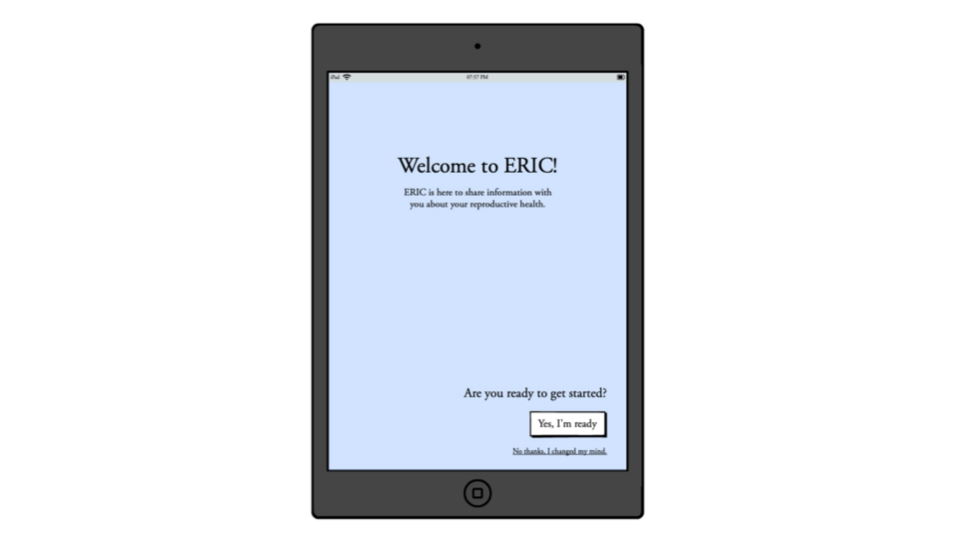
A screenshot of the initial prototype of the Dr. Eric intervention that was used by adolescent and young adults who were emergency department patients to provide feedback and usability information. Eric: Emergency Room Intervention to Improve Care.

This phase culminated in conceptualizing the brand and storyboard of the intervention, Dr. Eric, which connects to a sexual health program we formally developed and tested focused on adolescent female ED patients [24]. The brand of Dr. Eric is that of a reliable male medical provider who provides straightforward information about sexual health including condom use, effective female birth control methods, and communication strategies with partners regarding contraceptive use. The interviews discussed an initial low-fidelity prototype of Dr. Eric, as seen in Figure 3, built in Typeform.

### Phases 3 and 4: Design and Develop

#### Adolescent and Young Adult User Interviews

We conducted 8 adolescent and young adult interviews with baseline demographic data available for all 8 and interview transcripts available for 7. Participants ranged in age (14, 15, 17, 19, 19, 20, 20, and 21 years). The majority identified as Hispanic (7/8, 88%), having insurance (7/8, 88%), and being in high school or college (7/8, 88%), with many identifying as being of “other” race (3/8, 38%) or “not want to answer” (3/8, 38%) that question. Many answered that they had regular doctors (6/8, 75%); of those, some were spoken to about condoms (4/6, 67%). The average number of sexual partners in their lifetime was 4 (SD 1.5), with the median age of sexual intercourse being age 14 years. Four (50%) used a condom at the last intercourse, and most (7/8, 88%) had not talked to their current or last partner about pregnancy prevention. All had unlimited SMS texting plans on their mobile device, and most (7/8, 88%) had sole access to those texts. The following section details each module of the intervention with corresponding interview feedback.

#### Module 1: Goal Setting and Self-Reflection Around Family Formation

**Figure 4:**
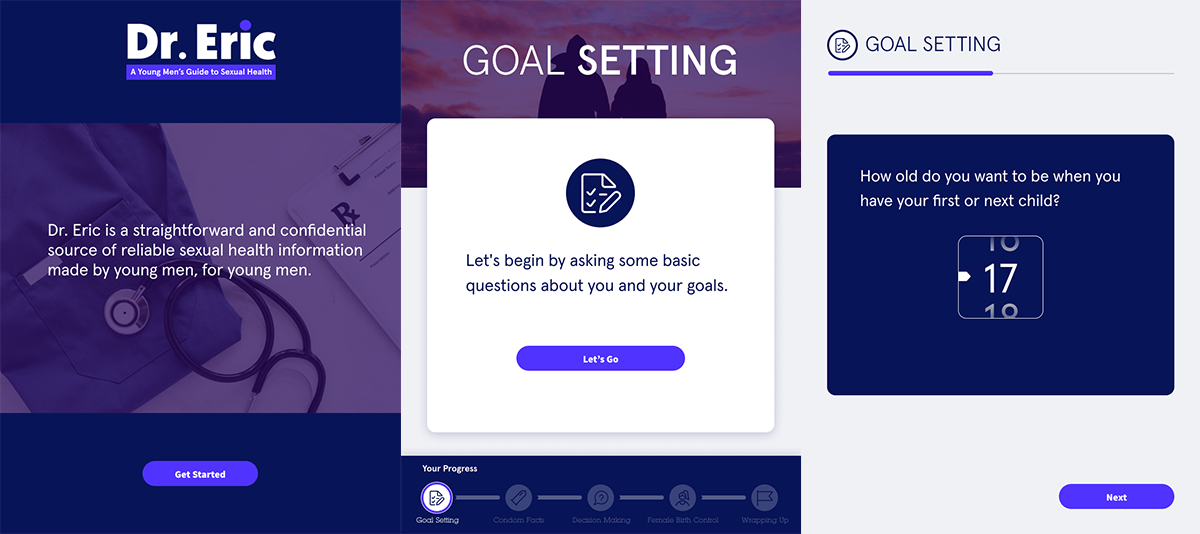
Screenshots of the landing page and first module, goal setting, of Dr. Eric app. Eric: Emergency Room Intervention to Improve Care.

The purpose of the first module was to support a user’s beliefs in his abilities to set goals around family formation and pregnancy prevention (self-efficacy) and affirm a sense of empowerment. The landing page of the app and the beginning of the first section can be seen in Figure 4. Adolescent and young adult users appreciated the straightforward tone of the Dr. Eric slogan, stating that “as soon as you open the app it tells you what it is and what it’s for.” While engaging with the first module, the app prompted users to answer goal-setting questions related to family planning or formation such as how old the patient would like to be when they have their first child. Users found that this section “gives you an option to open up and seek the help that you need.” Regarding the question asking about goals prior to having a child, one male remarked his appreciation for the question and stated, that even if he impregnated his partner, he liked knowing “that you still have that dream to do that accomplishment.” Users recommended the benefit of personalizing goal-setting questions to the geographical location; for example, “‘buy a home’ is a bit specific because we live in NYC...it suggests that if you want to build a family it’s preferred to have a home or a house.”

#### Module 2: Condoms

**Figure 5:**
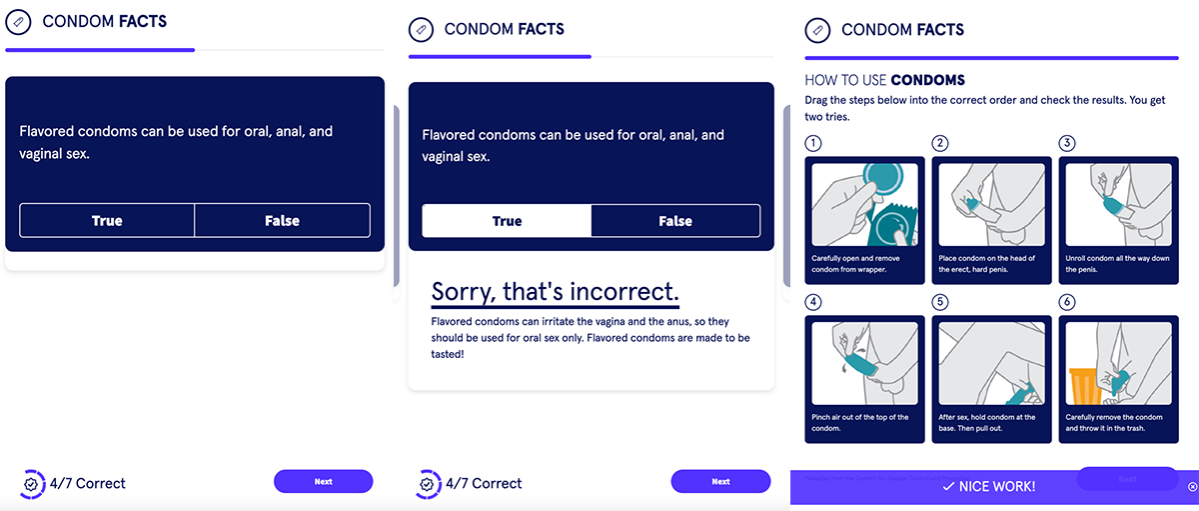
Screenshots of the second module of the Dr. Eric app that focused on condom use. Eric: Emergency Room Intervention to Improve Care.

The second module, “condoms,” as Figure 5 shows, intended to increase knowledge and skills regarding proper condom use. It started out with 6 true or false questions about condom use, which was then followed by a short video animation of how to use a male condom (sourced from the CDC). Participants were then asked to apply what was learned in the video and unscramble the chronological steps of using a male condom in a digital game (sourced from the CDC). When engaging with this section, adolescent and young adult users toggled between wanting this information to be presented in a straightforward, mature tone while also acknowledging the benefits of a comedic effect, such as condom icons. One user started laughing upon viewing images of condoms; when asked further, the user stated that, “it’s all about just getting out the laugh, I think that’s what most health classes are.” Regarding the true or false section, users stated the importance of the information being portrayed in a straightforward manner—“if you just sugarcoat things then what’s the point of just giving somebody [information].” In the same section, users enjoyed the “interactive” component of the module—“it feels like I’m playing a game and not that someone is just hitting facts at me.” Users wanted to learn “something new” in this section, as opposed to what is seen in “a lot of health classes”; there was a benefit to true or false questions that would “tell you the why,” as answers were linked to explanations. Further, users appreciated the “motivation” given by the “Nice Work!” banner on the screen when the order of condom use was finally correct.

#### Module 3: Decision-Making and Partner Communication

**Figure 6:**
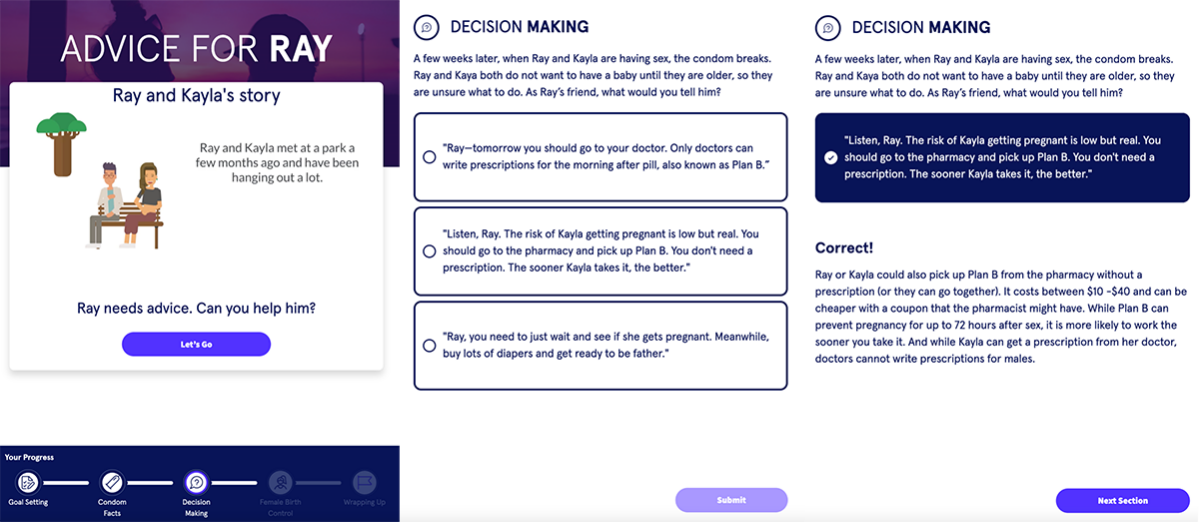
Screenshots of the third module of the Dr. Eric app that focused on decision-making and partner communication. Eric: Emergency Room Intervention to Improve Care.

The third module with example screenshots in Figure 6 aimed to incorporate a video story between 2 sexual partners representing the arc of an adolescent relationship in order to support partner communication, particularly around using birth control methods. The video frequently pauses, at which point the user is prompted with decision-making–style questions about how the character should proceed. Here, adolescent and young adult users noted the module’s benefits for patients who have “been in a similar situation,” emphasizing that it is “relatable.” One user remarked, “I like how it’s basically helping you put yourself in his shoes.” Other users were skeptical regarding receiving advice from friends, as opposed to experts. Furthermore, users “like [the module] pausing during the video” to ask questions, preferring this to watching the entire video and then answering questions. Some users suggested making the story more personalized, based on the goal settings inputted in the first module.

#### Module 4: Female Birth Control

**Figure 7:**
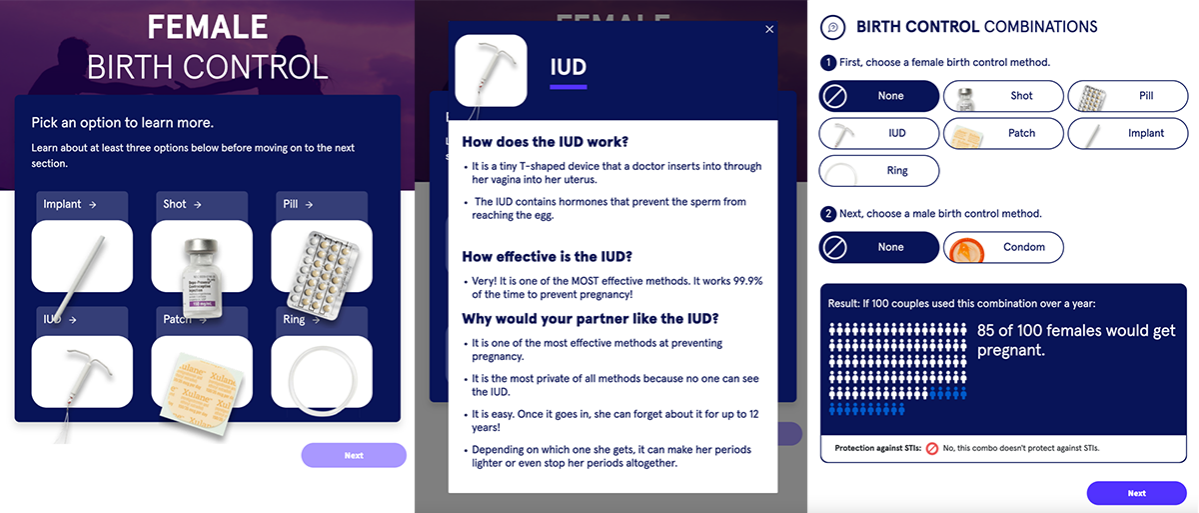
Screenshots of the fourth module that focuses on female birth control options, how they work, and their effectiveness at preventing pregnancy with or without a condom. IUD: intrauterine device.

The fourth module, as Figure 7 shows, prompted users to select multiple methods of female birth control to learn more about in subsequent panels. Then, users selected a combination of a female birth control method plus a male birth control method (with or without a condom); once the combination was clicked, a graphic illustrated the proportion of 100 female patients who would become pregnant in the subsequent year using those methods and whether or not the combination is protective against STI transmission using validated published quantitative algorithms [47]. Users were then asked their comfort level toward talking to partners regarding female birth control, which led them to a video demonstrating how to discuss safe sex with a partner to augment self-reflection. Adolescent and young adult users viewed this module as “a very simple way to educate people,” which one user viewed as necessary since “a lot of guys don’t feel the need to go and educate themselves on female birth control methods.” Users were surprised to learn of many different forms of birth control, stating “I learned something today...women do go through a lot, like a patch?!...a ring, how is that even put in?! What is an IUD?! All I know is the shot and the pill...see this is the reason why, see I could use this app.” They expressed a desire to learn more than surface-level information about the different methods of female birth control such as information regarding short- and long-term adverse effects on both the female and male, purpose and permanency of the method, and types of male birth control methods. Regarding the information presented, one user noted, “there’s a lot to learn about this because some don’t work, some people think can prevent [pregnancy], but you know stuff happens...I think this is a good topic.” Upon the completion of this module, users stated, “stuff like this will give you more chance to learn about something.” Users remarked upon the birth control combination portion of this module as helpful and descriptive; one user stated, “[this is] my favorite slide because it’s...choose this, choose this, and this is the result...cause and effect.” Users remarked upon the benefit of seeing the consequences of different combinations; “it’s actually pretty good...it’s telling people how if you don’t want to get pregnant at all, it tells you which is best to use.” Another user stated, “it’s good because it’s...showing you what choices you could make so you could be safe.”

#### Module 5: Goal Setting and Wrapping Up

**Figure 8:**
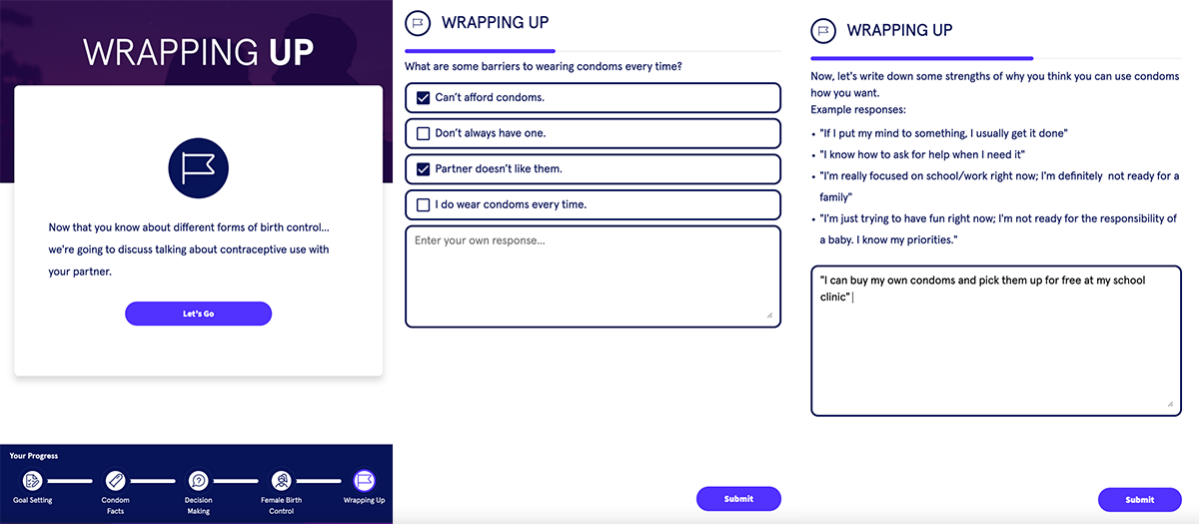
Screenshots of the final module of Dr. Eric app that presents open-ended questions about condom use and collects information for follow-up. Eric: Emergency Room Intervention to Improve Care.

In the fifth and final module, as Figure 8 shows, the Dr. Eric app posed thought-provoking questions that probed personal motivators and barriers to wearing condoms along with questions asking patients (1) to list their strengths that aid them in their goal of using condoms and (2) to assess their readiness to consistently use condoms. Finally, a short video summarized the next steps of the intervention, including how texting will be received and how to submit questions into the “LIVE Office Hours” platform. The penultimate screen led to self-refection by displaying the life goals they initially submitted in the first module, followed by a closing line that reads “You’re in control of your future. Use the knowledge you gained today to reach your goal.” Adolescent and young adult users described the purpose of the app as to “[tell] you what you need to do to feel comfortable.” Users went on to describe their perceived function of the app as a way to “just talk to teenagers basically and show them...what you need,” especially since “teenagers more likely...won’t talk to their parents about when they’re having intercourse, and they’ll keep it...a secret.” Users found encouragement in the app, stating, “knowing that you are the one that is in control...you control what will happen.” Other users interpreted this last slide as “reminding you that if anything happens to never forget your goals and to always be there thinking about them.” Additional suggestions included a comments section, setting up meetings, and creating a “Google classroom.” Adolescent and young adult users appreciated the ability to enter their own personal strengths, motivators, and barriers regarding wearing condoms and suggested “confetti or...a round of applause” to mark their completion of the modules. Upon completion of the app, one user stated, “it has helped a lot, it could teach people stuff that they don’t know...it has shown me different, some other things for birth control that I could use that I didn’t even know about.”

#### Text Messaging

**Figure 9:**
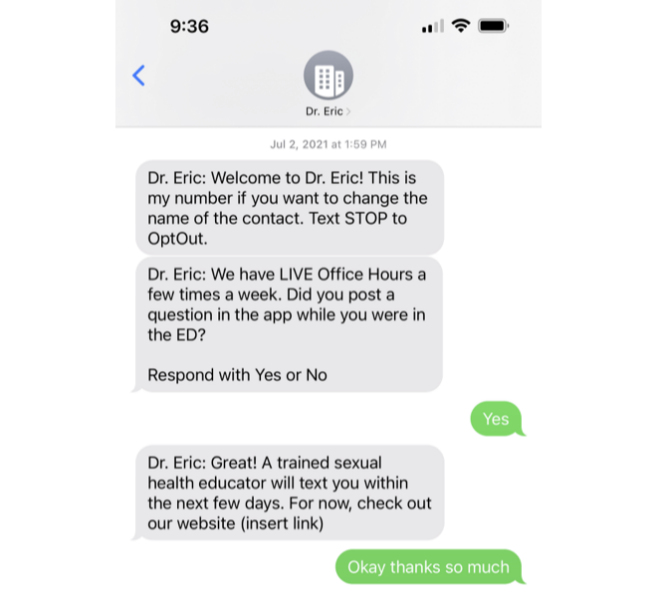
Screenshots of an example of a Dr. Eric SMS text messaging conversation. Eric: Emergency Room Intervention to Improve Care.

As the final component of the intervention, we wrote twelve 2-way, automated, SMS text messages to send weekly after ED discharge, which were personalized to participant baseline data and reinforced sexual health teaching points as in Figure 9*.* Adolescent and young adult participants advised the texts to be a source of “reminding you to always stay safe [during intercourse]...to always bring a condom with you and always stay protected.” Users thought patients would be more likely to ask personal questions in the app or “just through the text message.” Users suggested including information about additional topics not covered in the app such as HIV, pre-exposure prophylaxis, postexposure prophylaxis, sexual performance, and reproductive organs.

#### Informatic Experts Interviews

We interviewed 5 usability experts and organized their feedback and comments based on 8 of 10 heuristic principles that were relevant to our app. As seen in Table 1, we explain the definition of each principle along with exemplary quotes from experts. In addition to comments organized by these principles, usability experts also suggested clarification regarding where to click, changing wording and symbols to gamify the modules, developing “buttons” that transfer properly to iPads, and modifying wording to adequate literacy level. Finally, we assessed Dr. Eric using a mHealth checklist, which can be found in Multimedia Appendix 1.

**Table 1:** Nielsen’s heuristics with definitions and exemplary quotes from usability experts^a^.

Nielson’s heuristics	Exemplary quote from a usability expert
1. Visibility of system status	“It would be good to have some type of indicator on the screen...a progress indicator, because you don’t want them to feel like lost...when people feel lost, they just start ignoring the content.”
2. Match between system and the real world	“Based on my experience, they [adolescents] prefer to watch video compared to reading text. So, if that video includes all this information, probably, you don’t need to provide this as a text all here.”
3. User control and freedom	“The back button is important for the user’s control and freedom...they should be able to change their answer any time they want.”
4. Consistency and standards	“In the real-world setting, we usually use this clear box for the multiple-choice question. So, I’m not exactly sure about your study population, but I think, I guess, they are very familiar with a computer screen and this kind of things...once they see this clear box, they would think ‘I can’t select all that applies.’”
5. Error prevention	“So, this error message is because, I did it wrong?...Nice work. This error message, it looks good.”
6. Recognition rather than recall	“You want to make sure that people don’t have to remember, recognition rather than recall. So, the kind of thing where I might have to recall and that would be a reason I would go back, would be an example of when they are having a little quiz and you have circumvented that by bringing the question, by bringing my answer forward. So, I didn’t need to go back. To me, it’s not any kind of fatal flaw. I didn’t feel the need to go back and I think as long as you are clear that this is something that’s done in a sequence and when you’re finished with the section, you are finished with the section.”
7. Aesthetic and minimalist design	“And then, the information is here and it...it feels like a little text-happy to me...I just feel like in just looking at it, I’d say, boy, that’s a lot to read.”
8. Help and documentation	“With the FAQ style, what ends up happening is all of these heading that are listing here and you can click on them, and then you can actually have more choices at the top, but all this content is still down at the below, if people just decide to just scroll through and read everything...Then, you would then have to have some type of button here to go back to the top, like a finger pointing up or something, to go back to the list of questions.”

^a^Heuristic terms referenced from Nielsen [43,44]. Here, we describe 8 of 10 heuristics, as 2 are not relevant to this study.

## Discussion

### Principal Findings

The improvement of the sexual health of adolescents and young adults remains a national priority; yet, few theory-based, user-informed interventions focusing on adolescent and young adults and lead to lasting behavioral change and are implemented in locations where this at-risk population can be found. In this paper, we presented 4 iterative phases of digital intervention creation. Through a collaboration between an academic team and a digital design agency, we rigorously moved through each step of the process to define our population and problem; incorporate key stakeholder input, theoretical frameworks, and a logic model for change; build on patient personas and the ED experience; and refine low- and high-fidelity prototypes using the input of male ED patients and informatics experts. This led to the formation of an evidence-based multimedia program that aimed to improve the sexual health of patients who might not be receiving sexual health care elsewhere. These strategies can be used by other ED researchers interested in creating patient-centered, theory-based digital interventions.

### Comparison to Prior Work

Our findings add to the literature in 3 unique ways. First, the processes we created built on existing literature describing the iterative process needed to create effective mHealth interventions and added the novelty of developing it for the ED population and implementing it within an ED setting [25,26,48]. In our process, we focus on the distinctiveness of the acute care ED setting. Our model aimed to appreciate the mindsets and personas of users who may feel rushed, anxious, and even in pain. We appreciated distinct barriers that may oppose the implementation of the intervention and its fidelity and adoption, such as wireless disruptions if reliant on ED internet, the need for providers to prioritize patient flow, and provider lack of time to discuss preventive care not directly related to the patient chief complaint [36]. We also noted those facilitators that might enhance the engagement of the user with our intervention such as using the long wait time and lack of outside influences to distract the user. By interviewing patients during their ED visits, we obtained “in situ” feedback, meaning they interacted with app prototypes exactly where we intended to launch it [27]. This reinforced the potential success of the intervention both in its ability to engage the participant and exist within the ED space.

Our research focused on a complex population—adolescents. Researchers and companies creating adolescent-focused behavioral health interventions face overwhelming barriers [49]. Confidentiality can become jeopardized, particularly regarding sensitive issues including sexual and mental health, especially when mHealth apps collect data. Often the adolescent is forgotten in the development process of mHealth interventions, as it is harder to conduct user testing with those who cannot legally provide their own written consent with a parent. Most importantly, impacting adolescent and young adult behaviors proves extremely difficult, as they face a myriad of external factors consistently affecting their daily decisions and their health and well-being. Therefore, it is not surprising how adolescents as a class are often excluded from participation in clinical trials, studies in public health prevention, and other critical research efforts due to the complexity of obtaining informed consent while maintaining confidentiality [50]. The result is that treatment options and the design of interventions for adolescents must often be extrapolated from studies involving either children or adults. Yet, laws have evolved over time to try to include adolescents in research, given that adolescents show a significant ability to provide informed consent and possess the cognitive ability to make decisions about research participation, similar to these abilities in adults [51]. Beginning in the 1960s, laws in many states began to accord adolescents the right to consent to emergency care and medical treatment of conditions such as pregnancy, STIs, and drug, alcohol, and mental health problems. Nevertheless, these laws remain inconsistent between states, with adolescents requiring parental consent for contraception and other health services [52]. This need for consent often spills over to the research side, where to include a teenager in a sexual health study, one might need parental consent, which then risks the confidentiality of the adolescent. In this study, we were able to obtain a waiver of parental consent from our institutional review board and managed to create an app that aims to maintain privacy and engage the user.

Our third contribution to the literature is a detailed explanation of our partnership between an academic and business team and the unique ways we hybridized our models. By working together, we leaned on established behavioral science while leveraging commercial tools and techniques to develop a product on a budget while also using modern software development. Typical commercial development of mHealth apps often starts with the goal of helping users change established behaviors; however, the approaches often lack rigor and reproducibility [53,54]. Even basic usability testing, to make sure the app is operable to most users, may be omitted or minimized to conserve budget or release earlier [55]. For-profit software companies may focus on monetization as well as associated metrics like subscription purchases or cancellations. There may be a cost to maintain and develop new features, so monetization will often be the priority at the expense of the user experience. An example is subscription workflows; instead of allowing the user to cancel their account in 1 or 2 clicks, apps will often add additional required steps to make it more difficult for the user to finish the process [56]. Alternatively, a traditional academic or pharmaceutical approach to software development also presents shortcomings. While the efficacy of these apps, and whether or not they can create lasting or even temporary change, can be measured, organizations must collect substantial pilot data and receive considerable governmental grant funding to muster the resources and expertise to run clinical trials [57]. Objectively measuring the efficacy of a mHealth app requires lengthy studies, during which the app needs to remain relatively unchanged. This is inherently incompatible with modern agile software development, where apps are constantly being tested and iteratively improved to keep up with changing technologies and shifting market conditions [26]. Being able to quickly pivot strategies and deploy new features is critical for a chance at commercial success. The processes we describe in this paper aim to explain how we merged these methodologies.

Our findings also need to be situated in the context and location in which Dr. Eric was developed. Dr. Eric was created in the ethnic and cultural context make-up of New York City. This is particularly important, as discussions related to sexual health, whether it be on contraception or abortion, can be highly variable and often reliant on the dynamics in which they exist. The population seeking care in our urban ED identifies as mainly Hispanic and of mixed race; this finding is consistent across our pilot work and this study. Prior research explores how such cultural constructs as masculinity, sex roles, and religiosity may play a large role in decision-making around contraception among Hispanic male patients [58]. However, in interviews with our urban, Hispanic, English-speaking population from New York City, the factors influencing condom use focused more on a lack of knowledge of effective birth control options and skills to discuss them with partners and less on traditional Hispanic cultural-specific dynamics. One possible explanation for this may be the strength of New York City sexual health education and access to local sexual health services. While few states integrate policies to effectively incorporate comprehensive sexual health education into their curriculum, in New York City, students are required to have sexual health as part of the curriculum [59]. This may have led us to create an intervention starting from where the New York City schools left off, focusing on those domains our population felt they most needed to better understand.

This study also encourages us to think about how we might adapt Dr. Eric to other populations who may differ from the one we targeted. Future iterations of Dr. Eric must appreciate the local school-based and community-based initiatives that aim to improve sexual health and strategize how to work alongside such interventions that may vary based on the political or cultural environment in which they exist. To adapt Dr. Eric for other populations and the settings in which they live, additional usability testing would be required that explores app acceptability and how to tailor it for the population of interest [60]. Unique changes would have to be made that meet the sexual health needs of a variety of populations ranging from those who identify as gender minority or sexual minority or who may live in locations with school-based abstinence-only sexual health education.

### Limitations

First, we present our process to design Dr. Eric; more data are needed to determine its efficacy and acceptability among a large cohort of patients. Second, although we conducted qualitative interviews in our define phase with medical ED providers to understand strategies to implement preventive health interventions in the ED, questions were not specific to how Dr. Eric should be implemented. Third, despite funding for the project, the design of Dr. Eric was prohibited by cost; additional capital may have led to further rounds of iteration. Fourth, more data are needed to demonstrate how well Dr. Eric can implement into the existing ED system. Future work will focus on evaluating the efficacy of Dr. Eric via a randomized controlled trial and its implementation in the ED setting. In that trial, we will compare sexually active teens who meet study criteria (eg, medically stable and not cognitively impaired) and compare their condom use to those who do not interact with the Dr. Eric program.

### Conclusions

This paper details the process our multidisciplinary team took to create Dr. Eric, a novel, adolescent-informed, theory-based intervention promoting condom use and healthy sexual relationships built for adolescent and young adults in the ED setting. Given that EDs care for millions of adolescents and young adults each year, many with many unmet health needs, interventions are needed that aim to promote health equity and improve the health of patients where they seek care. By following the pragmatic steps outlined, other researchers can create ED-based digital interventions that deliver evidence-based education and support to ED patients.

## Appendix

Multimedia Appendix 1 Dr. Eric and the mobile health (mHealth) evidence reporting and assessment guidelines and checklist, including mHealth essential criteria. Eric: Emergency Room Intervention to Improve Care.

## Abbreviations

CDC: Centers for Disease Control and Prevention
ED: emergency department
Eric: Emergency Room Intervention to Improve Care
mHealth: mobile health
STI: sexually transmitted infection

## Data Availability

The datasets generated and analyzed during this study are not publicly available but are available from the corresponding author on reasonable request.
