# Supplementary material for: A Digital Sexual Health Intervention for Urban Adolescent and Young Adult Male Emergency Department Patients: User-Centered Design Approach
Source: JMIR Form Res. 2024 Oct 4;8:e55815. doi: 10.2196/55815 (PMC11489791; doi:10.2196/55815)
Supplement: Multimedia Appendix 1 [file formative_v8i1e55815_app1.pdf]

Criteria and Corresponding Responses:

1. Infrastructure (population level): Clearly presents the availability of infrastructure to support technology operations in the study location. This refers to physical infrastructure such as electricity, access to power, connectivity etc. in the local context.
Response: The tablet used for Dr. Eric connects to a secure wireless internet connection in our emergency department.

2. Technology platform: Describes and provides justification for the technology architecture. This includes a description of software and hardware and details of any modifications made to publicly available software
Response: The Dr. Eric intervention was built as a responsive web application and used JQuery and Bootstrap libraries to simplify the codebase.

3. Interoperability/ Health information systems (HIS) context: Describes how mHealth intervention can integrate into existing health information systems.
Response: The intervention was coded as a responsive web application. We also built a content management interface where the team can easily edit intervention content and see and export responses from users who went through the intervention. During this phase, we also implemented a short messaging service texting campaign run through an external company.

4. Intervention delivery: The delivery of the mHealth intervention is clearly described. This should include frequency of mobile communication, mode of delivery of intervention (that is, SMS, face to face, interactive voice response), timing and duration over which delivery occurred
Response: We designed and iterated the content of a two-part intervention—an app consisting of five educational modules, followed by 10 weeks of once-a-week interactive text messages. Participants took approximately 10 minutes to complete the app; the texts were sent weekly for 10 weeks.

5. Intervention content: Details of the content of the intervention are described. Source and any modifications of the intervention content is described.
Response: As part of a needs assessment, we collected data from: (1) key stakeholders (eg, high school teachers); (2) national and state sexual health curricula; (3) evidence-based sexual education guidelines; and (4) an extensive literature review, including experience implementing sexual health digital interventions in our ED. We chose the Social Cognitive Theory (SCT) and Motivational Interviewing (MI) to inform our intervention. This led to the design of a two-part intervention—an app consisting of five educational modules, followed by 10 weeks of once-a-week interactive text messages.

6. Usability/content testing: Describe formative research and/or content and/or usability testing with target group(s) clearly identified, as appropriate
Response: We conducted 8 user and 5 informatics expert interviews. After each round of testing, we analyzed feedback and made updates to the design and content of the prototype.

7. User feedback: describes user feedback about the intervention or user satisfaction with the intervention. User feedback could include user opinions about content or user interface, and their perceptions about usability, access, and connectivity.
Response: Interviews with eight AYA male ED patients suggested that users preferred: (1) straightforward information; (2) a clear vision of the purpose of Dr. Eric; (3) open-ended opportunities to explore family planning goals; (4) detailed birth control method information; and (5) interactive games presenting novel information with rewards. Five usability experts provided heuristic feedback aiming to improve the ease of use of the app.

8. Access of individual participants: Mentions barriers or facilitators to the adoption of the intervention among study participants. Relates to individual-level structural, economic and social barriers or facilitators to access such as affordability, and other factors that may limit a user's ability to adopt the intervention.
Response: We appreciated distinct barriers that may oppose the implementation of the intervention and its fidelity and adoption, such as wireless disruptions if reliant on ED internet, the need for providers to prioritize patient flow, and provider lack of time to discuss preventive care not directly related to the patient chief complaint. We also noted those facilitators that might enhance the engagement of the user with our intervention such as utilizing the long wait time and lack of outside influences to distract the user. Other barriers include privacy from family when completing the app.

9. Cost assessment: Presents basic costs assessment of the mHealth intervention from varying perspectives. This criterion broadly refers to the reporting of some cost considerations for the mHealth intervention in lieu of a full economic analysis. If a formal economic evaluation has been undertaken, it should be mentioned with appropriate references. Separate reporting criterion are available to guide economic reporting.
Response: Future iterations of Dr. Eric would require additional funding; to sustain Dr. Eric over time would also require additional funding.

10. Adoption inputs/ programme entry: Describes how people are informed about the programme including training, if relevant. Includes description of promotional activities and/or training required to implement the mHealth solution among the user population of interest
Response: In the next phase of this project, the testing of Dr. Eric in a randomized controlled trial, we will provide training to the providers who would be screening for Dr. Eric patients. Patients who interact with Dr. Eric will be provided more information about the app/study when recruited and consented for the study.

11. Limitations for delivery at scale: Clearly presents mHealth solution limitations for delivery at scale.
Response: More data are needed to determine Dr. Eric's efficacy and acceptability among a large cohort of males. Although we conducted qualitative interviews in our define phase with medical ED providers to understand strategies to implement preventive health interventions in the ED, questions were not specific to how Dr. Eric should be implemented.

12. Contextual adaptability: Describes the adaptation, or not, of the solution to a different language, different population or context. Any tailoring or modification of the intervention that resulted from pilot testing/usability assessment is described
Response: Future iterations of Dr. Eric must adapt to different populations including those who do not speak English. Future iterations must also appreciate the local school-based and community-based initiatives that aim to improve sexual health and strategize how to work alongside such interventions that may vary based on the political or cultural environment in which they exist. Unique changes would have to be made that meet the sexual health needs of a variety of populations ranging from those who identify as gender minority or sexual minority or who may live in locations with school-based abstinence-only sexual health education.

13. Replicability: Detailed intervention to support replicability. Clearly presents the source code/screenshots/ flowcharts of the algorithms or examples of messages to support replicability of the mHealth solution in another setting
Response: The program of Dr. Eric (app + text messages) can be experienced by participants in other settings as they are automated. However, before testing in another setting, more data is needed to demonstrate how well Dr. Eric can implement into the existing ED system. Future work will focus on evaluating the efficacy of Dr. Eric via a randomized controlled trial and its implementation in the ED setting. In that trial, we will compare sexually active teens who meet study criteria (eg, medically stable, not cognitively impaired) and compare their condom use to those who do not interact with the Dr. Eric program.

14. Data security: Describes the data security procedures/ confidentiality protocols
Response: In this research study, we obtained a waiver of parental consent from our IRB and built an app that aimed to maintain privacy.

15. Compliance with national guidelines or regulatory statutes: Mechanism used to assure that content or other guidance/information provided by the intervention is in alignment with existing national/regulatory guidelines and is described.
Response: The mobile platform provider conducted all texting dialogue compliant with HIPAA and the Code of Federal Regulations.

16. Fidelity of the intervention: Was the intervention delivered as planned? Describe the strategies employed to assess the fidelity of the intervention. This may include assessment of participant engagement, use of backend data to track message delivery and other technological challenges in the delivery of the intervention
Response: Dr. Eric was delivered as intended with few technological issues while evaluated in the ED during interviews. Future research is needed to determine its fidelity on a larger scale.

Adopted from Agarwal S, LeFevre AE, Lee J, et al. Guidelines for reporting of health interventions using mobile phones: mobile health (mHealth) evidence reporting and assessment (mERA) checklist. BMJ. 2016;352:i1174. doi:10.1136/bmj.i1174
